# Cross-cultural adaptation and validation of the 22-item sinonasal outcome test (SNOT-22) in German-speaking patients: a prospective, multicenter cohort study

**DOI:** 10.1007/s00405-021-07019-6

**Published:** 2021-08-05

**Authors:** Tobias Albrecht, Achim Georg Beule, Tanja Hildenbrand, Kathrin Gerstacker, Mark Praetorius, Claudia Rudack, Ingo Baumann

**Affiliations:** 1 Department of Otorhinolaryngology, Head and Neck Surgery, University Hospital Heidelberg, Medical Center-University of Heidelberg, Im Neuenheimer Feld 400, 69120 Heidelberg, Germany; 2 Department of Otorhinolaryngology, Head and Neck Surgery, Medical Center-University of Münster, Münster, Germany; 3 Department of Otorhinolaryngology, Head and Neck Surgery, Medical Center-University of Freiburg, Freiburg, Germany; 4 Department of Otorhinolaryngology, Head and Neck Surgery, University Medicine Greifswald-University of Greifswald, Greifswald, Germany; 5 Department of Otorhinolaryngology, Head and Neck Surgery, Medical Center-University of Hamburg, Hamburg, Germany

**Keywords:** Chronic rhinosinusitis, Health-related-quality-of-life, Patient reported outcome measures, QoL questionnaire

## Abstract

**Purpose:**

Chronic rhinosinusitis (CRS) is a common condition associated with a significant reduction of the health-related quality of life. One of the most widely used assessment tools in CRS is the disease-specific, health-related questionnaire SNOT-22. The aim of this study was to translate and validate the SNOT-22 into the German language.

**Methods:**

The questionnaire was translated using the forward–backward translation technique. After the translation its reliability, validity, and sensitivity were evaluated. For this purpose, the questionnaire was completed by patients diagnosed with CRS before, 3 months and 1 year after endoscopic sinus surgery and by healthy individuals as controls at three university hospitals in Germany. The individual scores of the questionnaire before surgery was correlated with the Lund–Mackay score as well as a global disease-specific question.

**Results:**

A total of 139 CRS patients and 31 healthy individuals participated in the study. Internal consistency at all timepoints was very good, with Cronbach’s alpha scores of 0.897, 0.941, and 0.945. The questionnaire was able to discriminate between CRS patients and control subjects (*p* < 0.0001) and scores improved significantly 3 month and 1 year after sinus surgery (*p* < 0.0001), indicating a good test–retest reliability, validity, and responsiveness. A significant correlation to the single global disease-specific question could be found (*p* < 0.0001), but no correlation with the Lund–Mackay score.

**Conclusion:**

The German Version of the SNOT-22 is a reliable, valid, and sensitive instrument for measuring health-related quality of life in patients with CRS. It can be recommended for clinical practice and outcome research for German-speaking patients.

## Introduction

Chronic rhinosinusitis (CRS) is a common condition worldwide, affecting 5–12% of the general population. This leads to a significant burden on society in terms of healthcare demands and productivity loss [1, 2]. The direct cost for the healthcare system in Europe are estimated at 1501€ per year in a cohort of patients with CRS with nasal polyps (CRSwNP) [3]. More important for patients suffering from CRS, however, it is associated with a significant reduction of the health-related quality of life, with a greater impact on social functioning than angina pectoris or chronic heart failure [4]. Chronic rhinosinusitis in adults is defined as an inflammation of the nose and the paranasal sinuses characterized by two or more symptoms for a period longer than 12 weeks. One of the symptoms should be either nasal blockage or nasal discharge in combination with facial pain/pressure and a reduction or loss of smell and either endoscopic sign of nasal polyps or mucopurulent discharge [5].

Health is a multidimensional concept, encompassing physical, mental, and social state of being [6]. The primary goal of medical treatment in chronic diseases is to achieve and maintain clinical control, which can be defined as a disease state in which the patient is asymptomatic or symptoms do not negatively affect quality of life (QoL) [5].

With the recent approval of monoclonal antibodies for the treatment of CRSwNP by the European Medicines Agency, a validated tool for assessing QoL is a prerequisite for adhering to the EPOS guidelines for their use [5].

One of the most widely used assessment tools in CRS is the disease-specific, health-related questionnaire SNOT-22. The questionnaire was initially developed and psychometrically validated in English language [7] and proved superior to 14 other QoL questionnaires for the evaluation of patients with CRS due to its reliability, validity, responsiveness, and ease of use as well as its high credibility for postoperative assessment [8]. The questionnaire covers 22 symptoms reflecting the health burden of the rhinological patient [7]. Each item quantifies the severity of the symptom from 0 (no problem) to 5 (worst possible symptom), resulting in a maximum total questionnaire score of 110. To date, it has been adapted and validated in several other languages [9–20]. The translation and validation of the questionnaire was initiated by the Working Group for Rhinology (ARHIN) of the German Society for Otorhinolaryngology, Head and Neck Surgery, to enable international multicenter studies including German study sites and to compare the burden of disease internationally. Permission for translation and cultural adaptation was obtained from the developer of the questionnaire Jay Piccirillo.

With this prospective, multicenter cohort study we aimed to culturally adapt the questionnaire into German and to evaluate its reliability, including the internal consistency and reproducibility, its validity and its responsiveness to treatment.

We intended to facilitate the applicability of the SNOT-22 in German-speaking patients, and to enable national, intercultural, and cross-country studies.

We hypothesized that the questionnaire could not only be translated and culturally adapted into German, but that the German translation would also have strong reliability as well as good validity and responsiveness.

## Materials and methods

### Translation and cultural adaptation

The translation and cultural adaptation of the SNOT-22 questionnaire was carried out in accordance with the guidelines and standards for the translation and cultural adaptation of Patient Reported Outcome measures as recommended by the ISPOR Task Force [21].

The original questionnaire was forward-translated by two independent German native-speakers with academic knowledge of English. The independent translations were then compared by two independent otolaryngologists, familiar with the process of instrument validation, and merged into a single forward translation, which was then translated back into English by two independent native English speakers with an academic German background. As a next step the back translated instruments were compared to the original questionnaire by two experienced rhinologists and the most appropriate alternatives were selected for each item.

As a last step of the procedure of translation and cultural adaptation, a representative group of patients (*n* = 15) with the diagnosis of a CRS, were enrolled in a pilot study at the University of Heidelberg to pre-test the questionnaire. Each patient autonomously completed the preliminary translated version of the SNOT-22 and discussed the wording and meaning of each item with the senior clinician to ensure the questionnaire was understandable and culturally meaningful. The suggested modifications of the wording were again discussed by the two experienced rhinologists. The questionnaire was modified on the basis of the suggestions resulting in the final version of the German SNOT-22 questionnaire. For the questionnaire all rights are reserved; Copyright 2006, The Washington University in St. Louis, Missouri.

### Patient recruitment

The study population included German native speaking adult patients of both genders, who met the EPOS 2012 criteria for chronic rhinosinusitis [22] and were able to give informed consent prior to a planned endoscopic sinus surgery (FESS).

Patients were prospectively recruited at the Department for Otorhinolaryngology of the University of Heidelberg, University of Freiburg and University of Münster. A total of 139 patients agreed to take part in the study and completed the questionnaire preoperatively. All patients were contacted 3 months and 1 year after the sinus surgery.

The control group consisted of 31 healthy volunteers of both genders, aged 18 years and above, that were randomly recruited among family members accompanying patients.

### Ethical considerations

The research protocol was approved by all three participating institutional ethics committees (University of Heidelberg, S-484/2015; University of Freiburg, 546/17; and University of Münster, AZ 2017-120-f-S). Informed consent was obtained from all study subjects and data gathered from the questionnaire as well as through medical records were collected in an anonymized database.

### Reliability

The reliability of a test indicates the degree of accuracy with which the tested characteristic is measured [23]. To assess the reliability, the internal consistency and the test–retest reliability were determined. Internal consistency was assessed using Cronbach’s alpha. Cronbach’s alpha estimates between 0.7 and 0.95 were taken as an indication of acceptable internal consistency [24, 25]. Test–retest reliability measures the stability of the responses to a questionnaire over a period of time in which symptoms are not expected to change. To assess the stability of responses, randomly selected patients were asked to complete the questionnaire again 4 weeks after the postoperative measurement.

### Validity

Validity of a test indicates how precisely the test measures what it claims to measure [23].

As criterion-related validity dimensions the discriminant and convergent validity were determined. To assess the discriminant validity, or known-groups comparison, the SNOT-22 scores of the 139 included patients affected by CRS were compared with the data obtained from the control group with asymptomatic individuals. To depict the convergent validity all 139 enrolled CRS patients were asked to state their impairment in quality of life with a single global disease-specific question assessing the severity of his/her disease at the timepoint they completed the SNOT-22 questionnaire for the first time. The options to answer the questions ranged from “No impairment” to “minor”, “moderate”, “strong”, and “very strong impairment”. The answers to the global question were correlated with the scores of the SNOT-22. In addition, each CT scan of the enrolled patients was scored according to the Lund–Mackay score [26].

### Responsiveness and sensitivity to change

The responsiveness of a questionnaire demonstrates its ability to detect a clinical relevant change in its score over time after a therapeutic intervention [27]. To assess responsiveness, all patients were contacted 3 months and 1 year after sinus surgery and asked to complete the questionnaire. The differences between the mean scores were evaluated.

Furthermore, the observed change was correlated to a single global disease-specific question assessing the severity of the disease as well as satisfaction with the surgical intervention.

The sensitivity to change describes the performance of the instrument in distinguishing a change in an individual from someone who has not changed [27]. To determine the sensitivity to change, the standardized response mean (SRM) and the effect size for the pre- and postoperative scores were calculated.

### Statistical analysis

Sociodemographic and clinical characteristics were analyzed using the Fisher’s exact test and independent sample *t* test. Cronbach’s alpha coefficient was calculated to asses internal consistency of the questionnaire. Test–retest reliability, which reflects long-term stability with repeated testing, was analyzed using the Spearman’s correlation coefficient. The intraclass correlation coefficient (ICC) with absolute-agreement, 2-way mixed-effects mode [28] was determined by correlating the measurements obtained 3 months and 4 months after sinus surgery.

To determine discriminant validity, data were analyzed using a nonparametric Mann–Whitney *U* test. The convergent validity was assessed correlating the results of the single global disease-specific question with the SNOT-22 scores using Pearson’s correlation coefficient. Responsiveness of the questionnaire was tested by comparison of the SNOT-22 scores of the CRS pre- and postoperative group via Wilcoxon’s matched pairs test. SRM was calculated by dividing the mean score change by the standard deviation of the change [27]. Based on Cohen’s rule of thumb for interpreting effect size statistics values < 0.2 are rated as a minor effect, ≥ 0.2 to < 0.5 as a small effect, ≥ 0.5 to < 0.8 as a medium effect and ≥ 0.8 as a major effect [29]. The effect size was calculated as the difference between the pre-treatment group mean minus the post-treatment group mean, divided by the standard deviation of the initial pre-treatment value.

A *p* value of < 0.05 was considered significant. Unless stated otherwise, all tests were two-tailed.

## Results

### Demographic characteristics

A total of 139 patients diagnosed with CRS and 31 healthy controls participated in the study.

After 3 months, the completely answered questionnaires of 113 (81.3%) patients and after 1 year, the completely answered questionnaires of 87 (62.6%) patients could be included in the analysis. All controls had a complete data set and could be included in the analysis.

Their demographic data and co-factors are summarized in Table 1. No significant differences were found between the two groups regarding age and marital status (*p* = 0.978 and *p* = 0.617). CRS patients lost to follow-up were not statistically different from those who returned completed questionnaires at all measurement time points in regard to the total score of the questionnaire at the pre-operative timepoint (*p* = 0.67), the gender (*p* = 0.466) and the marital status (*p* = 0.581).

**Table 1:** Demographic data and co-factors of 139 patients diagnosed with CRS and 31 healthy controls participated in the study

	Controls	Patients	*p* value
Total, no	31	139	
Age, mean (± SD), years	48,4 (± 15,2)	48,6 (± 15,5)	0.978
Gender, no (%)			0.044
Female	19 (61%)	55 (40%)	
Male	12 (39%)	84 (60%)	
Marital status			0.683
Married	21 (68%)	85 (63%)	
Single	10 (32%)	50 (37%)	
Smoking, no. (%)			
Never smoked		65 (50%)	
Quitted smoking		39 (28%)	
Smoker		28 (22%)	
Preoperative impairment in QoL, no. (%)			
No impairment		4 (3%)	
Low		11 (9%)	
Medium		46 (36%)	
Strong		45 (35%)	
Very strong		22 (17%)	

### Reliability

#### Internal consistency

The Cronbach’s alpha scores for the SNOT-22, representing the internal consistency, were 0.897 for the pre-operative assessment, 0.941 for the assessment 3 months after surgery, and 0.944 1 year after surgery, indicating high internal consistency.

#### Test–retest reliability

A total of 80 randomly chosen patients were asked to complete the questionnaire again 4 weeks after the 3 months postoperative measurement. Of these a total of 67 patients returned both copies. The Spearman’s correlation coefficient *ρ* was 0.861 (*p* < 0.0001), indicating high reliability of repeated measures. The intraclass correlation coefficient was 0.939 with a 95% confidence interval of 0.902 and 0.963, indicating an excellent reliability.

#### Discriminant validity

Discriminant validity was determined by comparing the value of the total scores of the CRS group with the values of the total scores of the control group. The mean score of the CRS group was 41.69 with a standard deviation of ± 19.25. The mean score of the control group was 10.10 with a standard deviation of ± 8.93. Median of the groups were 41.0 for CRS and 9 for the control group, respectively. We found a statistically highly significant difference between the two patient groups (*p* < 0.0001), reflecting a good discriminant validity.

#### Convergent validity

The correlation of the single global disease-specific question and the SNOT-22 scores of the patient group, using Pearson’s correlation coefficient found a positive significant correlation with a correlation coefficient *r* = 0.504 (*p* < 0.0001). No significant correlation was found between the SNOT-22 scores and Lund–Mackay scores (*p* = 0.1239).

#### Responsiveness of the questionnaire and sensitivity to change

**Fig. 1:**
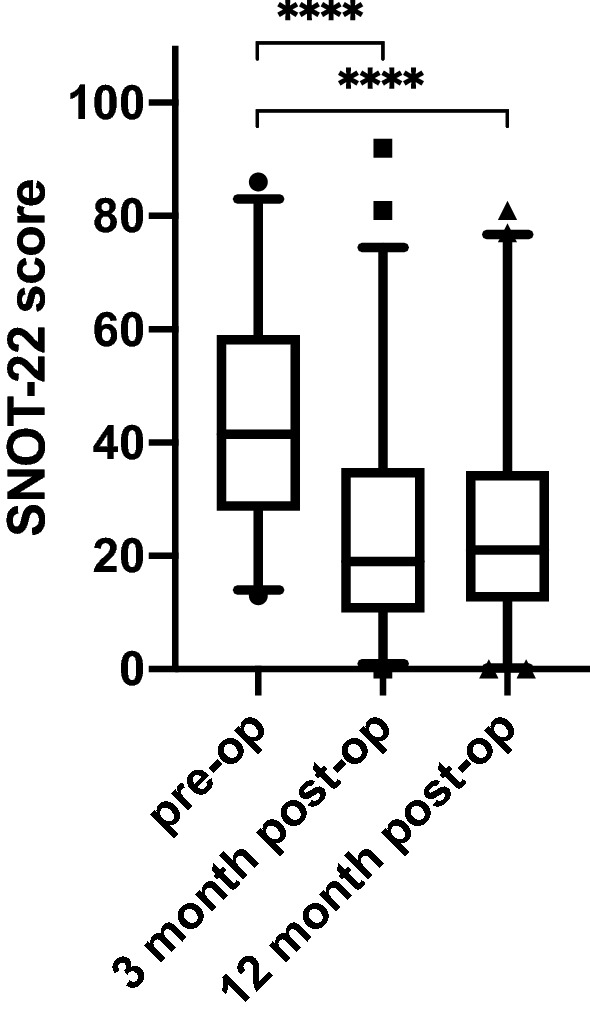
Total SNOT-22 scores preoperatively, 3 months and 12 months post-operatively. Whiskers indicate the 2.5% quantile and the 97.5% quantile. ****p = 0.0001

SNOT-22 scores of 106 patients affected by CRS were obtained 3 months after surgical treatment and compared to their pre-operative scores for responsiveness analysis. The SNOT-22 scores of 87 patients could be included 1 year after surgery. The results are presented in Fig. 1. In detail, the SNOT-22 scores obtained at the pre-treatment timepoint were significantly higher, with a mean value of 42.76 with a standard deviation of ± 18.91 and a median of 41.00, than those obtained 3 months after surgical treatment with a mean value of 25.9 and a standard deviation of ± 20.77 (*p* < 0.0001). 1 year after surgery the mean value was 25.54 with a standard deviation of ± 19.08 (*p* < 0.0001). Analysis of the pre-operative scores and the scores obtained 3 months after surgery revealed a mean score change of 16.87 with a standard deviation of ± 18.59. The pre-operative scores compared to the scores 1 year after surgery revealed a mean score change of 16.45 with a standard deviation of ± 20.24. The SRM and the effect size for the pre-operative and the postoperative timepoints revealed a major effect with an SRM of 0.91 and an effect size of 0.89 for the 3 months period and 0.81 and 0.87 for the 1 year period, respectively.

## Discussion

The SNOT-22 questionnaire is a validated assessment tool that is recommended for evaluating quality of life in patients affected by CRS [5]. This questionnaire has already been translated and adopted into different cultural and linguistic contexts.

In this study, the SNOT-22 was translated and adapted into German language by following the principles of good practice for the translation and cultural adaptation process [21]. This method ensures equivalence of the translated version to the original questionnaire and allows a comparison of responses. The results of the psychometric validation were comparable to the original validation of the questionnaire as well as the existing translations. To validate the reliability of the questionnaire the internal consistency and the test–retest reliability were calculated. Cronbach’s alpha between 0.70 and 0.95 indicates a good internal consistency for health questionnaires [25]. The internal consistency of the German Version of the SNOT-22 appeared to be good, with a Cronbach’s alpha of 0.89 in a group of 139 patients affected by CRS. This finding is comparable to other existing language translations, as the mean score for Cronbach’s alpha of 15 versions of the SNOT-22 is 0.88 with a minimum of 0.8 in the Arabic [10] and a maximum of 0.96 in the Moroccan translation [30]. A recent publication from Austria which validated but did not translate or adapt the German SNOT-22, also showed a comparable result of 0.93 for internal consistency [31]. An overview of the results of the different SNOT-22 translations is indicated in Table 2. The test–retest reliability was excellent, with an intraclass correlation coefficient of 0.939 and a correlation of *ρ* = 0.861, showing a high reliability of the questionnaire. The results for the test–retest reliability were also similar to the previous studies which had correlation coefficient values between 0.64 and 0.99 with a mean score of 0.86. The test–retest reliability was not investigated in the Austrian validation study. As the original questionnaire and the existing translations, the German version of the SNOT-22 questionnaire can reliably discriminate between healthy subjects and CRS patients (*p* < 0.0001). In contrast to some other translations, our mean scores of the CRS patients and the control group are almost identical to the scores of the original questionnaire.

**Table 2:** Overview of the results of the different SNOT-22 translations

Language	Internal consistency	Test–retest	Validity	Responsiveness	Mean score	
					CRS	Controls
Arabic [10]	0.803	0.907	< 0.001	< 0.001	64.2	19.5
Brazilian Portuguese [32]	0.88	0.91	< 0.0001	< 0.0001	62.4	11.4
Czech [18]	0.9	0.86	NA	NA	38.5	13.7
Danish [19]	0.83	0.7	NA	NA	29.7	NA
English [7]	0.91	0.93	< 0.0001	< 0.0001	42.0	9.3
French [9]	0.93	0.78	< 0.0001	< 0.0001	41.0	8.3
**German**	**0.897**	**0.861**	** < 0.0001**	** < 0.0001**	**41.69**	**10.1**
German (Austria) [31]	0.93	NA	< 0.001	< 0.001	38.0	15.1
Greek [14]	0.84	0.91	< 0.0001	< 0.0001	49.6	13.0
Hebrew [15]	0.94	0.88	< 0.0001	< 0.001	50.4	13.2
Italian [12]	0.86	0.85	< 0.008	< 0.001	48.9	14.3
Lithuanian [16]	0.89	0.72	< 0.0001	< 0.0001	52.4	16.8
Moroccan [30]	0.968	0.993	< 0.0001	< 0.0001	50.4	14.5
Russian [11]	0.816	0.98	< 0.0001	< 0.0001	67.6	9.3
Spanish [17]	0.91	0.87	< 0.0001	< 0.0001	47.2	4.5
Thai[33]	0.94	0.64	NA	NA	38.2	NA
Turkish [34]	0.88	0.97	< 0.0001	< 0.0001	64.3	15.6

Internal consistency shown as Cronbach’s alpha coefficient, test–retest reliability shown as Spearman’s or Pearson’s coefficient or ICC, validity shown as Student’s *t* test or Mann–Whitney *U* test, responsiveness shown as Student’s *t* test or Mann–Whitney *U* test

The bold values are the values of this manuscript. This should help the reader to find the new values and to compare them to the other publised values

They are also comparable to the results of the French translation, which is the translation to a language of a country in a very close geographical relationship with Germany [7, 9].

The differences in measurements in the different countries could be explained by several demographic confounders and comorbidities [35] or could indicate a cultural difference in the perceived impairment in quality of life due to the disease. The slightly weaker results of the Austrian validation of the German SNOT-22 in terms of validity and responsiveness could also be due to demographic confounders or to the fact that the German spoken in Austria is only similar to the German spoken in Germany, as the authors themselves admit [31]. Similar to the findings in the original study by Hopkins, the German SNOT-22 demonstrated an excellent correlation to the single global disease-specific question (*p* < 0.0001), further supporting the criterion validity of the questionnaire. Similar to previous reports, but unlike the Austrian validation, no significant correlation could be found between the SNOT-22 and the Lund-Mackay scores [9, 12, 31]. This underlines the fact, that it is the patient with his or her perceived impairment that should be treated and not the radiological image.

The large effect size of 0.89 shown by comparing pre-operative scores with the scores 3 months after surgery indicates, that FESS is an effective treatment option for the population studied. Encouragingly, the mean values of the SNOT-22 obtained at 1 year after surgery remained stable to the scores obtained 3 months after surgery, indicating a long-term effect of the treatment.

### Strength and limitations

In this study, all results of the psychometric validation of the German Version of the SNOT-22 were comparable to the original validation of the questionnaire [7]. The data shown thus allowed the SNOT-22 to be fully validated in a large study population recruited from three different university hospitals in Germany. However, this study has some limitations. Even though the control group matched the age of the CRS group, the gender distribution of the two groups differed. The control group was predominantly female, while the CRS group was predominantly male. A comparison of the control group with the patient group with regard to smoking was not possible, as the ratio of smokers was not recorded in the control group.

The study population is limited to a sample selected for FESS. CRS patients without planned surgery were not included.

Comorbidities such as allergy, aspirin intolerance, asthma or depression were not recorded, which are likely to be higher in patients with CRS [36] and could have affected the SNOT-22 score of the study population [37].

## Conclusion

The German version of the SNOT-22 is a reliable, valid, and sensitive instrument for measuring health-related quality of life in patients with chronic rhinosinusitis. It is characterized by its ease of use by patients and physicians. The present study facilitates the application of the questionnaire in German-speaking patients and enables the systematic inclusion and comparability of quality-of-life measures in international trials.
